# Label‐free multiphoton excitation imaging as a promising diagnostic tool for breast cancer

**DOI:** 10.1111/cas.15428

**Published:** 2022-06-22

**Authors:** Takahiro Matsui, Akio Iwasa, Masafumi Mimura, Seiji Taniguchi, Takao Sudo, Yutaka Uchida, Junichi Kikuta, Hidetomo Morizono, Rie Horii, Yuichi Motoyama, Eiichi Morii, Shinji Ohno, Yasujiro Kiyota, Masaru Ishii

**Affiliations:** ^1^ Department of Immunology and Cell Biology Osaka University Graduate School of Medicine Osaka Japan; ^2^ WPI‐Immunology Frontier Research Center Osaka University Osaka Japan; ^3^ Yokohama Plant Nikon Corporation Yokohama Japan; ^4^ Department of Hematology and Oncology Osaka University Graduate School of Medicine Osaka Japan; ^5^ Integrated Frontier Research for Medical Science Division, Institute for Open and Transdisciplinary Research Initiatives (OTRI) Osaka University Osaka Japan; ^6^ Laboratory of Bioimaging and Drug Discovery, National Institutes of Biomedical Innovation, Health and Nutrition Osaka Japan; ^7^ Breast Oncology Center, Cancer Institute Hospital of Japanese Foundation for Cancer Research Tokyo Japan; ^8^ Department of Breast Surgical Oncology New Tokyo Hospital Chiba Japan; ^9^ Division of Pathology Cancer Institute, Japanese Foundation for Cancer Research Tokyo Japan; ^10^ Department of Pathology Saitama Cancer Center Saitama Japan; ^11^ Department of Pathology Osaka University Graduate School of Medicine Osaka Japan

**Keywords:** artificial intelligence, breast cancer, multiphoton excitation microscopy, rapid diagnosis, surgical margin

## Abstract

Histopathological diagnosis is the ultimate method of attaining the final diagnosis; however, the observation range is limited to the two‐dimensional plane, and it requires thin slicing of the tissue, which limits diagnostic information. To seek solutions for these problems, we proposed a novel imaging‐based histopathological examination. We used the multiphoton excitation microscopy (MPM) technique to establish a method for visualizing unfixed/unstained human breast tissues. Under near‐infrared ray excitation, fresh human breast tissues emitted fluorescent signals with three major peaks, which enabled visualizing the breast tissue morphology without any fixation or dye staining. Our study using human breast tissue samples from 32 patients indicated that experienced pathologists can estimate normal or cancerous lesions using only these MPM images with a kappa coefficient of 1.0. Moreover, we developed an image classification algorithm with artificial intelligence that enabled us to automatically define cancer cells in small areas with a high sensitivity of ≥0.942. Taken together, label‐free MPM imaging is a promising method for the real‐time automatic diagnosis of breast cancer.

## Graphical abstract

Label‐free multiphoton excitation imaging is a promising method for the real‐time automatic diagnosis of surgical margins, which should be useful for intraoperative rapid analysis during breast‐conserving surgery.

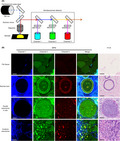

**Abbreviations**
AI: artificial intelligence
CNN: convolutional neural network
MPM: multiphoton excitation microscopy
ROI: region of interest
SHG: second harmonic generation

## 1 INTRODUCTION

Histopathological diagnosis has been the most important and definitive method of final diagnosis for over a century. With stained tissue preparations and bright‐field microscopy, pathologists estimate or judge different types of histopathological findings such as cell malignancy, the degree of inflammation and fibrosis, the depth of tumor invasion, and metastasis status. Moreover, histopathology plays an important role in the final diagnosis and intraoperative rapid diagnosis, which is critical for reducing the excision range in cancer surgery.^1^, ^2^, ^3^ However, some problems are associated with typical histopathology‐based diagnostic procedures. Conventional methods can provide only planar two‐dimensional images; thus, it is not possible to observe three‐dimensional structures in diverse anatomical structures consisting of various cells. In addition, it is difficult to analyze gene copy counting accurately using conventional methods by in situ hybridization because of thin tissue sections. Therefore, it would be desirable to develop an alternative diagnostic method that provides histological information in real time without tissue sampling.

Recent advances in optical imaging technology have enabled observing biological phenomena in real time. Multiphoton excitation microscopy (MPM) is one of the most common tools for intravital imaging. It enables the three‐dimensional observation of tissues within living animals in real time.^4^, ^5^, ^6^, ^7^ Several intravital imaging techniques use genetically fluorescent‐labeled animals; however, label‐free imaging with MPM can be performed using nonlinear optical phenomena and autofluorescence.^8^, ^9^ Recently, several reports have been published on novel imaging technologies applied to clinical medicine.^10^, ^11^, ^12^ Visualization of multiple areas in real time without tissue sampling and staining is likely to be advantageous in clinical histopathological diagnosis. For instance, the application of the aforementioned imaging technology may enable us to quickly grasp cellular or molecular features during surgeries.

In the present study, we aimed to establish a novel MPM imaging method for human fresh breast tissue, without fixation or staining. Furthermore, we also intended to describe the utility of our attempt to classify cancerous or noncancerous images using artificial intelligence (AI) based on a convolutional neural network (CNN). The combination of our MPM imaging and CNN‐based classification may be a promising alternative method with quantifiability and rapidity that can omit tissue sampling.

## 2 MATERIALS AND METHODS

### 2.1 Clinical specimens

Breast tissue samples consisting of 30 carcinoma tissues and 25 normal tissues were collected postoperatively from 32 patients with breast carcinoma from the Cancer Institute Hospital of the Japanese Foundation for Cancer Research (Table 1). Samples were collected from the center of the tumor or the residual normal breast tissue surrounding the tumors. They were immediately immersed in phosphate‐buffered saline with 10% fetal bovine serum and penicillin/streptomycin and delivered to the imaging room at Osaka University. Immediately following imaging, the samples were fixed in 10% neutral buffered formalin (Muto Pure Chemicals) and processed routinely for paraffin embedding. All patients were histologically diagnosed preoperatively using biopsies. All patients provided written informed consent in accordance with the ethics committee requirements of the hospital and the Declaration of Helsinki. This study was conducted with the approval of the ethics boards of the Cancer Institute Hospital of the Japanese Foundation for Cancer Research and the Osaka University Graduate School of Medicine. The Osaka University Graduate School of Medicine Institutional Review Board approved the study protocol on December 17, 2015 (No. 15369). The Cancer Institute Hospital of the Japanese Foundation for Cancer Research Institutional Review Board approved the study protocol on August 18, 2016 (No. 2016‐1059).

**TABLE 1:** Patients and tissue samples participating in this study

Characteristics	
Age at operation (years); mean (range)	56.6 (33‐77)
<50	13 (40.6%)
50–70	14 (43.8%)
≧70	5 (15.6%)
Patients and provided tissues (*n* = 32)	
Both normal and tumor tissues	23 (71.9%)
Only normal tissue	2 (6.3%)
Only tumor tissue	7 (21.8%)
Tissue size (mm)	
Normal tissue (*n* = 25)	
Major diameter (mean ± SD)	19.5 ± 5.00
Minor diameter (mean ± SD)	13.6 ± 3.66
Tumor tissue (*n* = 30)	
Major diameter (mean ± SD)	20.8 ± 5.64
Minor diameter (mean ± SD)	14.6 ± 3.86
Histological type of cancer (*n* = 30)	
Invasive breast carcinoma of no special type	28 (93.4%)
Mucinous carcinoma	1 (3.3%)
Invasive micropapillary carcinoma	1 (3.3%)

Abbreviation: SD, standard deviation.

### 2.2 Imaging of human breast tissues and group separation of data files

The imaging system consisted of an upright microscope (A1RMP+; Nikon) driven by a laser (Chameleon Vision II Ti: Sapphire; Coherent, Inc.) tuned to 780 nm, and an upright microscope equipped with a × 25 water immersion objective (CFI75 Apo 25 × W MP/NA 1.10; Nikon). Tissue samples were positioned with the observation surface facing upward and overlaid with a coverslip to place a drop of water between the sample and the objective lens. To detect multiphoton excited fluorescence and second harmonic generation (SHG) emission signals, we used 390/18‐nm, 480/40‐nm, and 629/56‐nm band‐pass filters. To acquire a series of image files from one sample, we collected image stacks at 3‐μm vertical steps at a depth > 30 μm below the sample surface with 1.0× zoom and 1024 × 1024 X‐Y resolution (0.50 μm per pixel). Following imaging, we calibrated the *z*‐coordinate (orthogonal to the observation plane) of the stacked image files by defining *z* = 0 as the first height at which the fluorescent signal was detected in almost the entire image area. Moreover, images of the surface side with *z* ≤ 30 μm were used in this study.

In normal tissues, the adipose tissues, fibrotic stromal tissues, and vascular structures were observed in addition to the ductal‐lobular structures. In cancerous tissues, we could recognize invasive breast carcinoma, mucinous carcinoma, and ductal carcinoma in situ in addition to the surrounding normal tissue components. An experienced pathologist linked the ground truth to each image file. We divided all image files from normal and cancerous tissues into two groups, depending on the case. Image data of group A (1765 images from 12 patients; consisting of 784 images from normal tissues, 91 images without malignant findings from cancerous tissues, and 890 images with malignant findings from cancerous tissues) were used for the construction of classification models, including training and validation. We used the training data set for the construction of the learning model candidates, and the validation data set was used to select the learning model with a high generalization performance. Lastly, we analyzed the classification performance of the determined learning model using the files of group B (2393 images from 20 patients, consisting of 899 images from normal tissues, 138 images without malignant findings from cancerous tissues, and 1356 images with malignant findings from cancerous tissues).

In the spectral analyses, we used the spectral detector of the A1RMP+ microscope (Nikon). Tissues were imaged to record their emission spectra from 380 nm to 630 nm (collected in 25 bins, each approximately 10 nm wide) under 780 nm excitation. Each fluorescence intensity was recorded at 4096 gray intensity levels (12 bits).

### 2.3 Construction of image classification model

All image files, in the form of squares with a side of 1024 pixels, were normalized before the analysis such that the mean value was 0 and the standard deviation was 1. Moreover, they were cropped into 64 squares with sides of 128 pixels (termed “image tiles”). After cropping, we separated the validation data set by randomly selecting 20% of the image tiles from group A; the resulting 90,368 and 22,592 tiles were prepared for training and validation, respectively. Subsequently, the image tiles with a signal‐to‐noise ratio < 20 and an average brightness value <0.10 were defined as blank. The signal‐to‐noise ratio was based on a pseudo‐noiseless image, which was constructed with a median filter. Based on these criteria, 9908 tiles in the training set, 2498 tiles in the validation set, and 12,258 tiles in the test set (group B) were judged as blank. After separating the blank image tiles, we established a classification model for the nonblank image tiles using Inception‐v3, a type of deep‐learning architecture. The code was implemented in Python using TensorFlow 2.0, and Keras 2.3, which are open‐source software libraries for machine intelligence. The Adam optimization algorithm was used with an epoch of 100, a learning rate of 0.020 and a batch size of 128. A total of 80,460 and 20,094 image tiles from group A were used for training and validation during training, respectively. Following the construction, all 140,894 nonblank image tiles from group B were tested for an automatic classification of the presence of cancer cells. After the test, all image tiles from group B (153,512 tiles), involving those judged as blank (12,258 tiles), were evaluated to calculate detection accuracy. The image tiles judged as representing cancer were classified as “positive.” In contrast, image tiles judged as showing no cancer cells as well as the blank ones were classified as “negative.”

### 2.4 Histology

Paraffin‐embedded specimens were cut into 4‐μm‐thick sections and stained with hematoxylin and eosin (H&E) using a standard protocol.

### 2.5 Statistical analyses

For comparisons between groups with non‐Gaussian distributions, we performed the Mann–Whitney *U* test to calculate *P* values. Accuracy represents the number of correctly classified observations over the total analyzed data number. Precision represents the ratio of correctly predicted positive observations to the total predicted positive observations. Recall, a synonym for sensitivity, represents the ratio of correctly predicted positive observations to all observations in actual positivity. F‐measure represents the weighted average of precision and recall. To compare diagnosis accuracies by experienced pathologists between conventional histopathological method using HE staining and MPM images, Cohen's kappa coefficient was calculated as a measure of interinspection reproducibility.

## 3 RESULTS

### 3.1 Autofluorescent profiles of human fresh breast tissues

**FIGURE 1:**
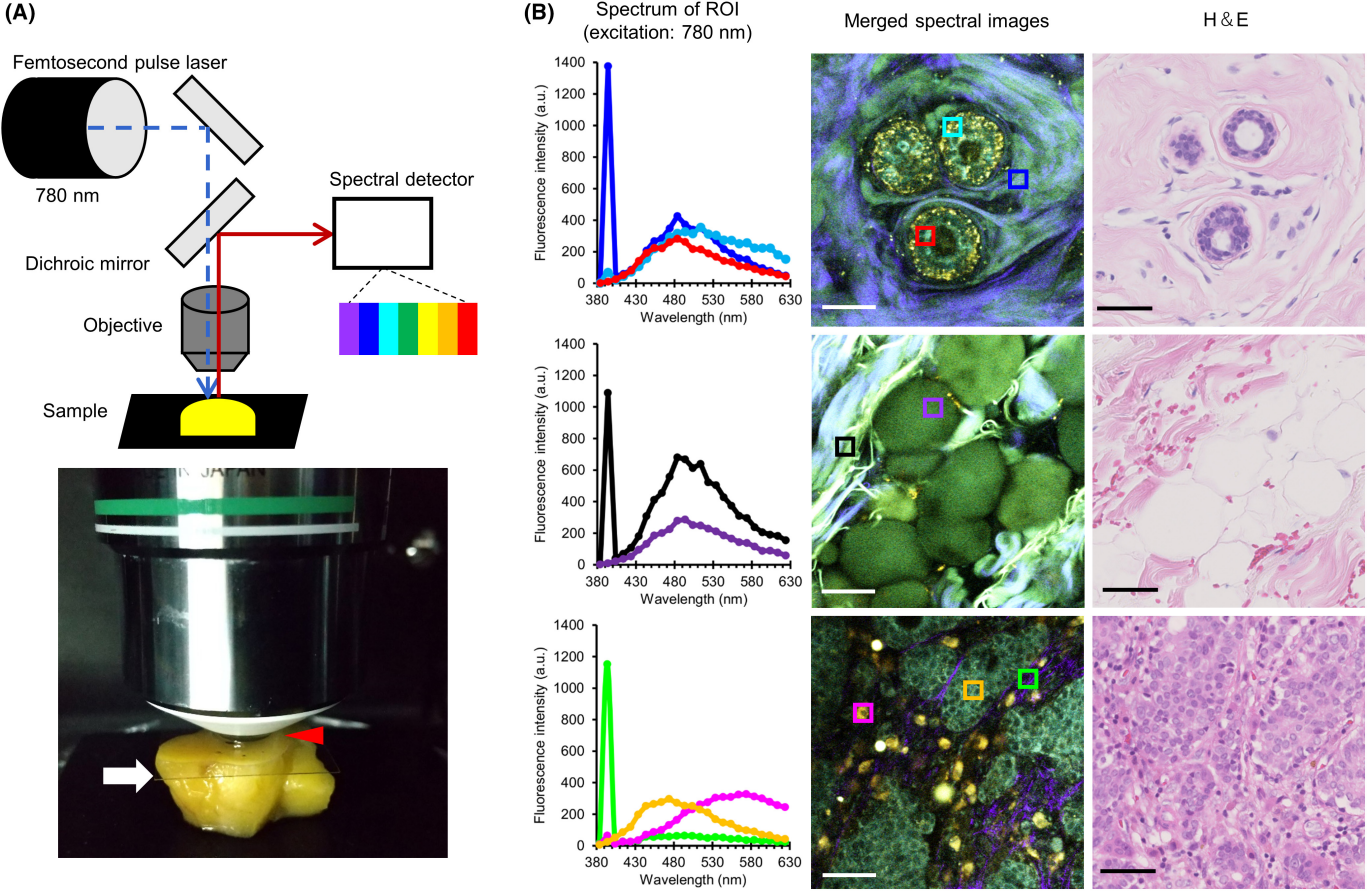
Spectral analysis of fresh human breast tissue with near‐infrared ray excitation. A, A schematic of spectral analysis using multiphoton excitation microscopy system. A coverslip (white arrow) is placed to retain water (red arrowhead) between the fresh tissue and objective lens. The excitation near‐infrared ray is tuned to 780 nm and emitted from the femtosecond pulse laser, and tissues are imaged to record emission spectra from 380 nm to 630 nm (collected in 25 bins, each approximately 10 nm wide). B, Representative results of spectral analysis using normal mammary gland tissue (first row), normal fat tissue (second row), and breast carcinoma tissue (third row). The color graph in the first column displays the fluorescence intensity at a similar color region of interest (ROI) (a square with 15 μm side) as in the middle column images. The images in the middle column consist of 25 image superpositions (described in Figure S1A–C). The hematoxylin and eosin (H&E)‐stained images in the third column are captured after the spectral analysis from the same tissue. Bar, 50 μm

We analyzed the autofluorescent spectral profile of fresh human breast tissues (Figure 1A). Previous studies using human fresh tissues other than breast reported on detecting sufficient autofluorescent signals by an excitation wavelength ≤800 nm.^12^, ^13^ Therefore, we employed an excitation wavelength of 780 nm in the current study. We continuously acquired emission signals between 380 nm and 630 nm under the aforementioned conditions (Figure 1B and Figures [Link], [Link]). Epithelial cells in the normal mammary duct displayed an emission peak at 480 nm (red region of interest [ROI] in Figure 1B). Similar autofluorescence was observed in adipose cells around the mammary gland (purple ROI in Figure 1B) and fibrous structures (blue and black ROIs in Figure 1B). We could also detect a dot‐like autofluorescence near the edge of normal ducts with an emission peak at 510 nm (cyan ROI in Figure 1B). In addition, we detected a sharp and narrow emission fluorescence peak at 390 nm from the fibrous structures (blue and black ROIs in Figure 1B). This represented half the excitation wavelength, thereby indicating that we observed fluorescent signals by SHG derived from fibrillar collagens.^14^ In cancerous tissues, the emission peak of cancer cells was similar to that of normal epithelial cells (orange ROI in Figure 1B). Contrarily, immune cells around the cancer cells displayed autofluorescence with an emission peak at 570 nm (magenta ROI in Figure 1B). Thin fibrous structures near the cancer cells emitted less autofluorescence, except for SHG (green ROI in Figure 1B). Therefore, human breast tissue emitted multiple autofluorescent signals via near‐infrared ray excitation. It principally consisted of three major peaks, which prompted us to describe the histological features in detail with these fluorescent signals.

### 3.2 MPM images of breast tissue enabled pathologists to estimate normal or cancerous lesions accurately

**FIGURE 2:**
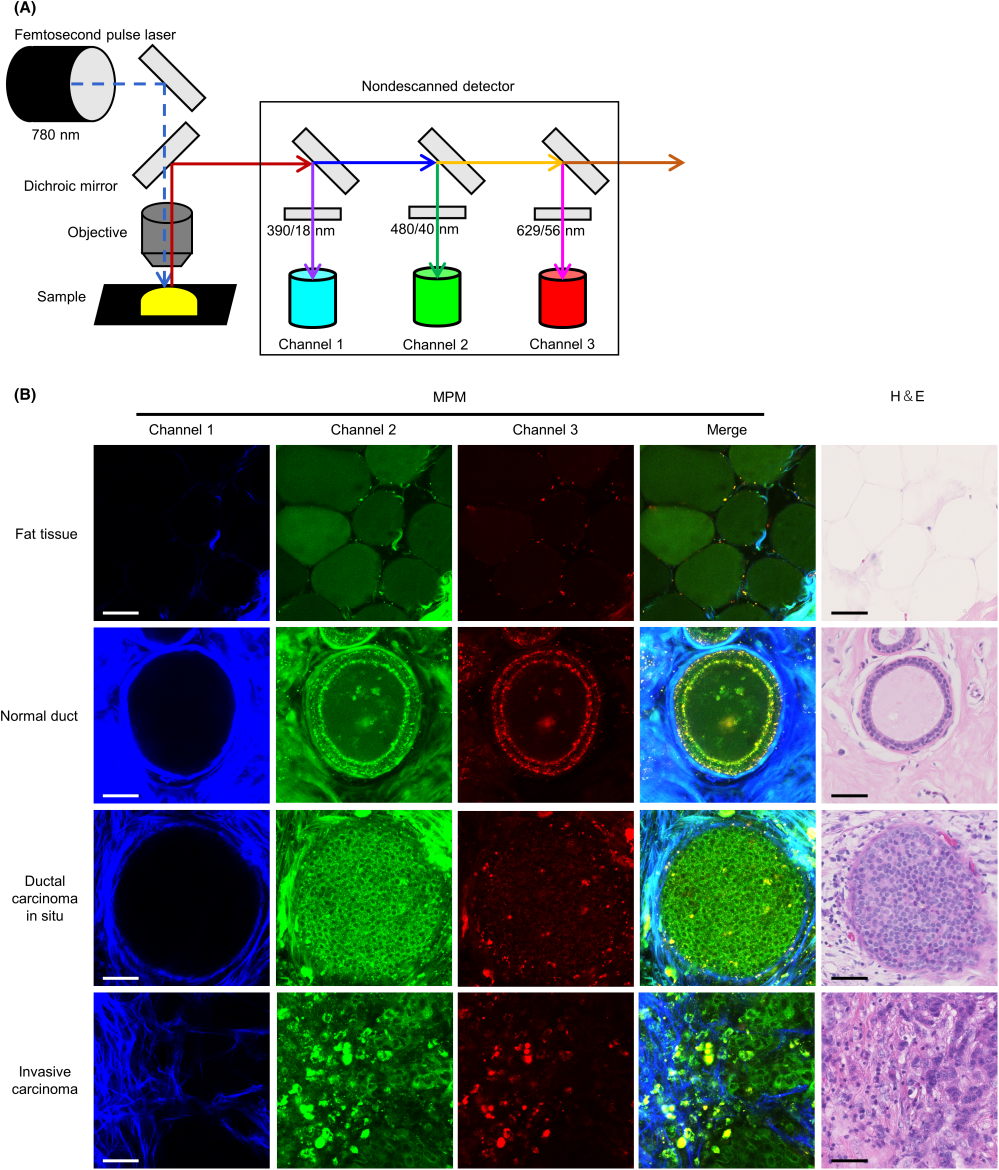
Label‐free multiphoton excitation microscopy (MPM) imaging for human fresh breast tissues. A, A schematic of the MPM imaging system. The excitation near‐infrared ray is tuned to 780 nm and emitted from the femtosecond pulse laser, and fluorescence signals are detected with nondescanned detectors after the transmission of dichroic mirrors and emission filters. B, Representative images of MPM imaging of breast tissue with various histological types. Merged fluorescent images (fourth column) are constructed by the superposition of three fluorescent images from different channels. Hematoxylin and eosin (H&E)‐stained images in the fifth column were captured after the imaging analysis from the same tissue. Bar, 50 μm

Based on the abovementioned spectral analysis, we established an MPM imaging system using three channels with different band‐pass filters (Figure 2A). Next, we performed label‐free MPM imaging of fresh unstained breast tissues, including normal and cancerous lesions. H&E‐stained sections from identical tissues used for MPM imaging were used to confirm the diagnosis. In fat tissues around the mammary gland, we identified adipose cells with circular morphology by detecting autofluorescent signals (first row of Figure 2B). Epithelial cells were lined neatly on the basal side of the lumen in mammary ducts in normal tissues. Cellular nuclei were identified as signal‐void regions and did not display obvious enlargement. In addition, dot‐like autofluorescence was detected along the edge of the ductal structures (second row of Figure 2B). In contrast, tumor cells of ductal carcinoma in situ revealed irregular proliferation in the lumen with nuclei enlargement on MPM imaging. Moreover, the dot‐like autofluorescence at the lesion edge was unclear (third row of Figure 2B). In invasive carcinoma tissues, the tumor cells proliferated irregularly without luminal formation (fourth row of Figure 2B). These histological features recognized in MPM images resembled those of conventional H&E‐stained images (fourth column of Figure 2B). Multiphoton excitation microscopy images of both normal tissues (from 25 patients) and cancerous lesions (from 30 patients) were randomly examined by two experienced pathologists, who could provide correct diagnoses using only the MPM images with a kappa coefficient of 1.0 (Table 2). These results demonstrated that experienced pathologists can estimate normal or cancerous lesions using only our MPM images with adequate high accuracy.

**TABLE 2:** Comparison of diagnostic results by pathologists between multiphoton excitation microscopy (MPM) images and conventional histopathological method using hematoxylin and eosin (H&E) staining

		H&E		
		Normal	Carcinoma	Total
MPM	Normal	25	0	25
	Carcinoma	0	30	30
	Total	25	30	55

### 3.3 Image classification with AI algorithm enabled to detect cancer lesion in small area without pathologist's intervention

**FIGURE 3:**
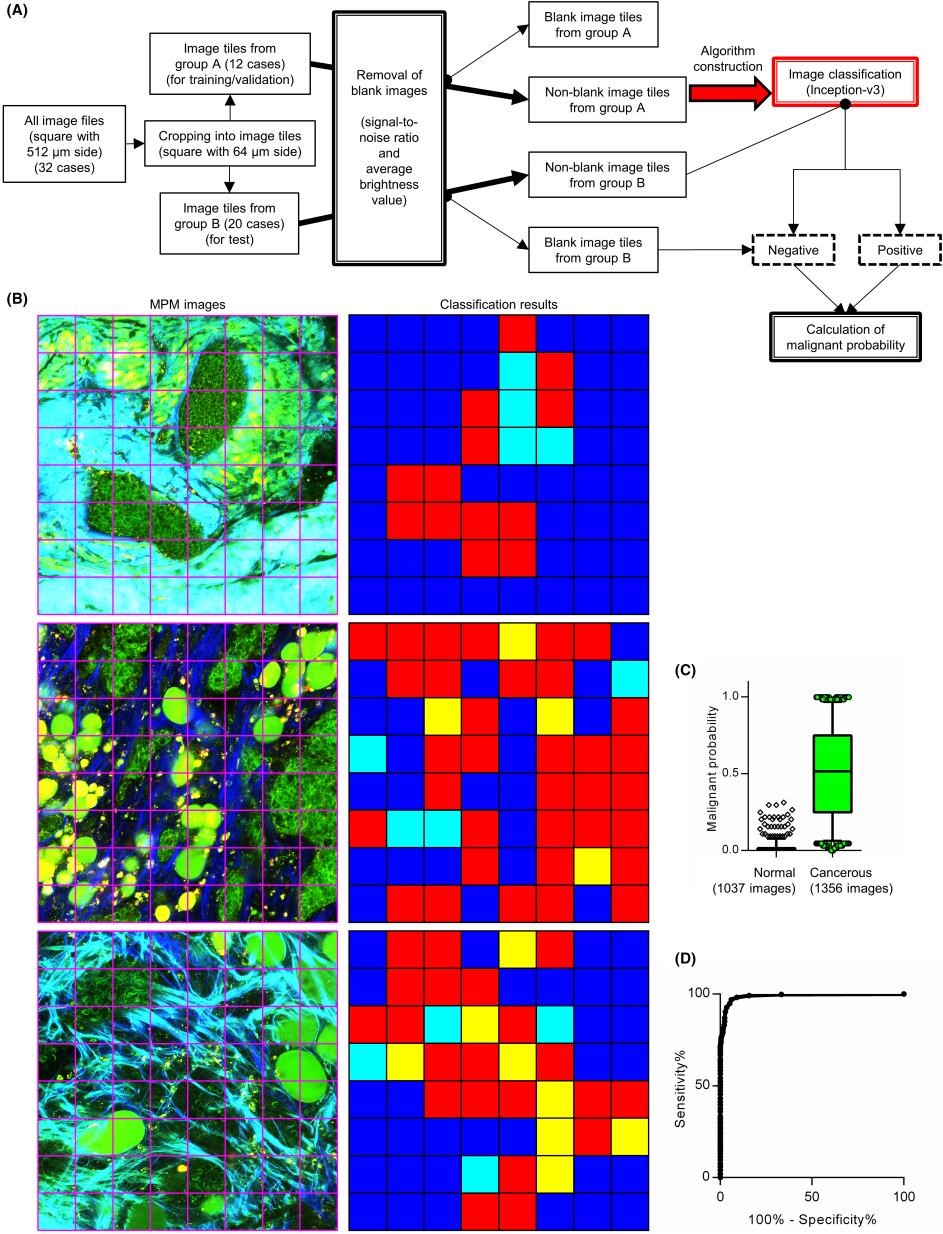
Multiphoton excitation microscopy (MPM) image classification algorithm to detect cancer cells in a small area using convolutional neural network (CNN)‐based artificial intelligence (AI). A, Flowchart of the classification algorithm. B, Representative classification results of the algorithm for each image tile (a square with 64 μm), cropped from the original image file (left column). Each square in the right column displays the results and corresponds to the image tile in the left column. Red, yellow, cyan, and blue indicate true‐positive, false‐positive, false‐negative, and true‐negative results, respectively. C, Box‐and‐whisker plot of malignant probability of each MPM image from group B. The top and bottom of the rectangle indicate the third and first quartile, respectively. Horizontal lines in the rectangle indicate the median. Vertical lines indicate the 5‐95 percentile range. D, Receiver operating characteristic (ROC) curve of malignant probability

When inspecting a small lesion or tumor margin, cancer cells are usually found only in a small area (if any). Therefore, we constructed an AI classification algorithm that could detect cancer cells that exist in a portion of the captured image (Figure 3A). We randomly divided all image files into two groups, depending on the patients. Subsequently, we cropped all files into 64 squares with 64 μm side each, or “image tiles,” and a pathologist linked ground truth to each tile. Considering the inclusion of blank image tiles in the edge area of the original images, we separated them based on the signal‐to‐noise ratio and average brightness value. Nonblank tiles from group A were used to construct an image classification model based on Inception‐v3, a type of CNN architecture. We used image tiles from group B to examine the classification algorithm. Both image tiles judged as involving no cancer cells as well as blank ones were classified as “negative.” In contrast, those judged as involving cancer were classified as “positive.” The utility test of our classification algorithm, performed with 153,152 tiles from group B, indicated accuracy, precision, recall, and F‐measure of 0.934, 0.827, 0.942, and 0.881, respectively (Figure 3B and Table 3). Moreover, to evaluate the detection accuracy of the malignant area in the original captured image file (consisting of 64 tiles), we defined the index termed “malignant probability” as the ratio of positive tiles in an original captured image. This index was calculated for each analyzed image file from group B, which revealed a significant difference between the normal and cancerous images (Figure 3C). Under the threshold of 0.0625 (from the receiver operating characteristic curve in Figure 3D), we classified 1314 of 1356 cancerous files, and 974 of 1037 normal files were classified as “malignant” and “nonmalignant,” respectively, with a sensitivity, specificity, and area under the curve of 0.969, 0.939, and 0.989, respectively (Figure 3D). These results indicated that a combination of label‐free MPM imaging and AI‐based image classification enabled the detection of cancer cells in a small area.

**TABLE 3:** Confusion matrix of multiphoton excitation microscopy (MPM) image classification algorithm for all image tiles from group B

		Actual value		
		Positive	Negative	Total
Predicted value	Positive	37,267	7815	45,082
	Negative	2283	105,787	108,070
	Total	39,550	113,602	153,152

## 4 DISCUSSION

In this study, we proposed the potential usability of MPM imaging for histopathological diagnosis of breast cancer. We consider this imaging method has three main advantages. First, it enables two‐dimensional observation with depth. We can evaluate only a single plane with conventional histology, while previous report using tissue‐clearing technique has indicated that three‐dimensional pathological screening might increase diagnostic sensitivity.^15^ In MPM imaging, it is possible to observe at different focal planes by “optical slice” and add higher dimensional information to conventional histopathology. Second, MPM imaging is superior in rapidity based on less invasiveness. As it does not require sampling or staining for observation, it is unnecessary to perform fixation, embedding, thin slicing, and staining. Such a time‐saving effect may be advantageous for intraoperative rapid diagnosis. Lastly, one of the most important features of MPM imaging is the easy access to the AI‐based image classification model. It is possible to obtain the digital image data from fresh tissue in real‐time. Thus, AI can be accessed rapidly and easily, without any image digitization process. Recently, the application of AI to histopathology has progressed remarkably and is now used in research and clinical practice.^16^ Multiple reports have suggested that CNN‐based AI analysis excels in image recognition or identification, and CNN algorithms can accurately analyze or classify histological images.^17^ Despite the problem of “explainability” of algorithm results,^18^ utilizing AI for histopathological assessment has several advantages.

Novel optical technologies other than MPM are also considered useful for histopathological analysis (Table 4). For instance, photoacoustic imaging is based on a hybrid technique of optical excitation and ultrasound detection and collects ultrasonic signatures from the expansion of molecules following laser light irradiation.^19^, ^20^ However, we cannot directly visualize cancer cells in label‐free conditions but rather judge them indirectly from the state of the tumor environment. Moreover, ultrasound has an inferior spatial resolution to light, and the in‐plane spatial resolution of photoacoustic imaging (approximately 150 μm^20^) is not sufficient to distinguish a lesion at the single‐cell level. Raman spectroscopy is another imaging device that detects the inelastic scattering of monochromatic laser light to probe molecular vibrations that provide a specific signal of cells and tissues.^19^, ^21^ However, it takes considerable time during measurement compared with other intravital imaging techniques. This is because Raman‐scattered light is faint. Taken together, the greatest advantage of the MPM imaging method is its ability to rapidly provide image data similar to conventional histological images at high resolution on a cell‐by‐cell basis.

**TABLE 4:** Comparison of multiphoton excitation microscopy (MPM) and other optical technologies for intraoperative diagnosis

	Photoacoustic imaging	Raman spectroscopy	MPM
Measured energy	Ultrasound	Raman scattered light	Visible light and near‐infrared ray
Analysis time	Seconds	Minutes to hours	Seconds
Tumor cell detection	Contrast agent	Label‐free (by Raman shift)	Label‐free (by autofluorescence)
Compatibility with conventional histology	Good	Not good	Good

We assume that our MPM imaging method is promising, especially in intraoperative rapid diagnosis. Although it is critical to reduce the excision range in cancer surgery,^1^, ^2^, ^3^ suboptimal accuracy is one of the most important concerns. This is partly attributed to sampling error caused by the limited observable area of the frozen section. Moreover, tissue image quality obtained from frozen sections is generally lower than that from permanent specimens because of the artifacts caused by freezing or compression.^22^ As for breast cancer, surgical margin diagnosis in breast‐conserving surgery displays inadequate sensitivity (ranging from 65.0% to 96.0%), compared with its specificity (ranging from 84.0% to 100%).^23^ In other words, the current methods may be insufficiently reliable for determining the margin status,^24^ and we require alternative methods to improve accuracy. Our MPM imaging may complement the conventional intraoperative rapid diagnosis and improve the rapidity. In current intraoperative diagnosis takes approximately 20 minutes from tissue sampling to slide glass preparation. In contrast, a series of our imaging procedures in this study has implied that multi‐layered MPM imaging of fresh tissue can be completed within at most 5 minutes by optical slices, and it takes only a few seconds to analyze the imaging data with AI, though supporting data about examination time are currently not available. Therefore, MPM imaging may shorten the examination time during surgeries. In addition, we can perform MPM imaging analysis ahead of the conventional method with frozen sections, which can be added afterward depending on the AI classification result. To verify these contents, it is necessary to reorganize the study for intraoperative diagnosis and compare the diagnostic accuracy as well as the actual time required for diagnosis.

This study has some limitations. Only surplus surgical tissues were analyzed in our study as we needed to collect fresh tissues before fixation and embedding. However, the study design restricted the collection of histological samples rarely found in surgical specimens. It was also impossible to collect particular histological images in advance unless it was recognizable by the gross appearance on fresh tissue. In contrast, the mammary gland tissue has a divertical histological appearance. Therefore, images from normal tissues analyzed in this study reflect only a small portion of non‐neoplastic breast tissues, while several other important tissue images are not included, such as atrophic ducts or lobules, adenosis, fibrocystic changes, and usual ductal hyperplasia. Similarly, we could not assess columnar cell lesions and premalignant lesions such as atypical ductal hyperplasia, which might be challenging in intraoperative rapid diagnosis. It is necessary to collect abundant image data that reflect various mammary gland histology, including biopsy specimens by multicenter analysis.

## DISCLOSURE

S.O. and E.M. are editorial board members of Cancer Science. The other authors declare no competing interests.

## ETHICS STATEMENT

All patients provided written informed consent in accordance with the ethics committee requirements of the hospital and the Declaration of Helsinki. This study was conducted with the approval of the ethics boards of the Cancer Institute Hospital of the Japanese Foundation for Cancer Research (No. 2016–1059, approved on August 18, 2016) and the Osaka University Graduate School of Medicine (No. 15369, approved on December 17, 2015).

## Supporting information


Figure S1

Figure S2

Figure S3
