# Supplementary figures and images for: Label‐free multiphoton excitation imaging as a promising diagnostic tool for breast cancer
Source: Cancer Sci. 2022 Jun 22;113(8):2916–25. doi: 10.1111/cas.15428 (PMC9357641; doi:10.1111/cas.15428)

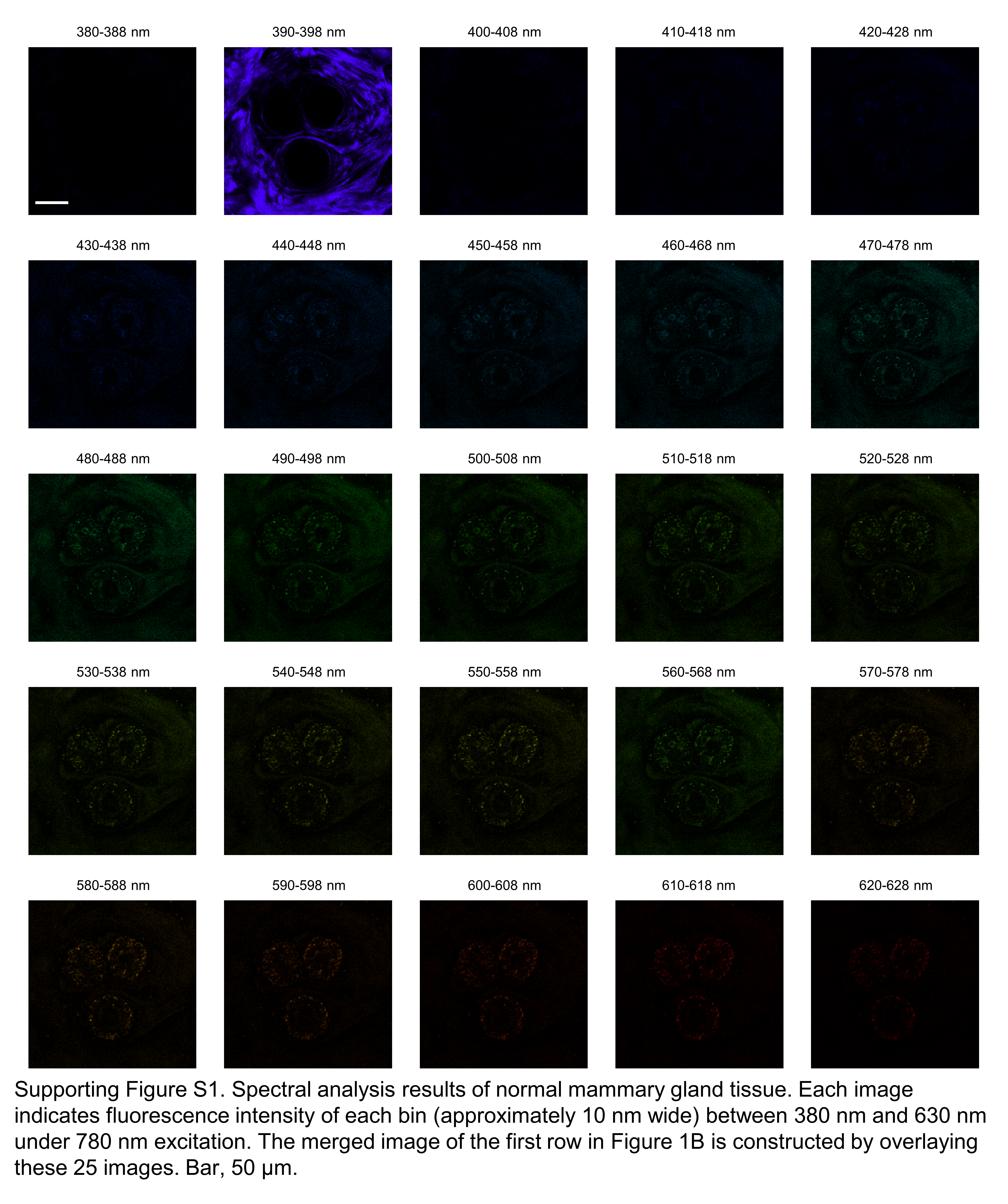

Supplement: Supplementary file 1 — Figure S1 [file CAS-113-2916-s002.tif]

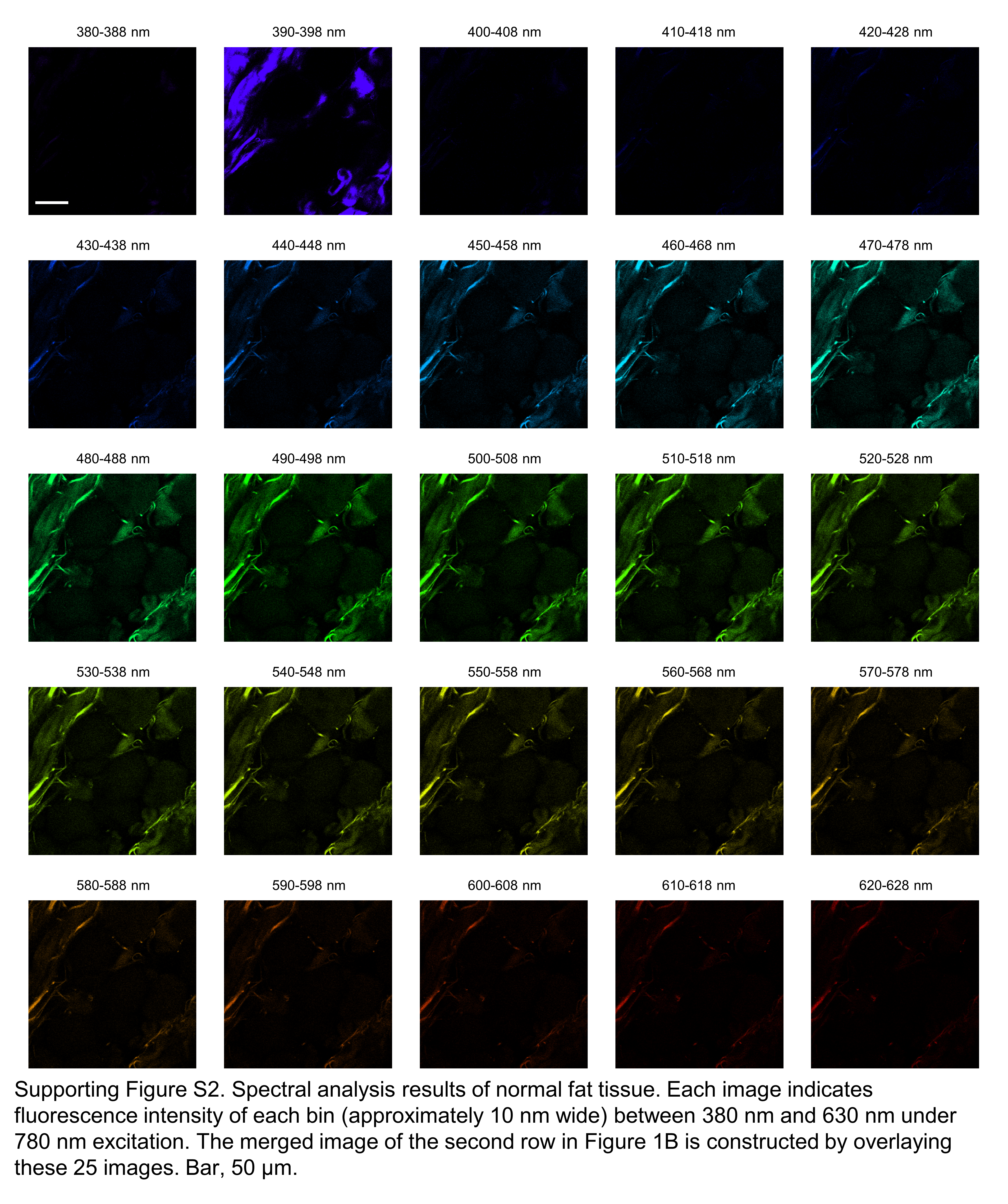

Supplement: Supplementary file 2 — Figure S2 [file CAS-113-2916-s003.tif]

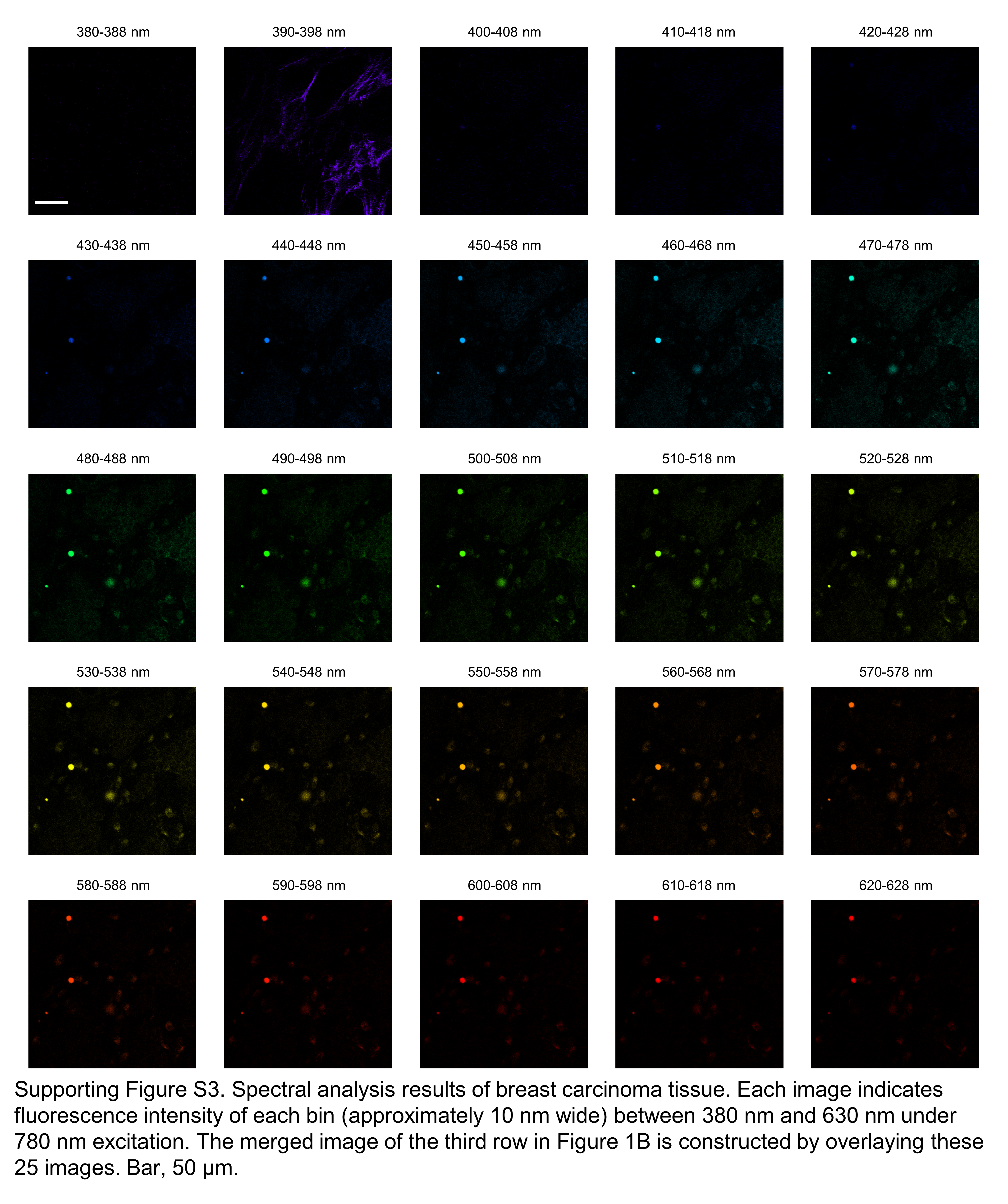

Supplement: Supplementary file 3 — Figure S3 [file CAS-113-2916-s001.tif]
